# Eating disorders amongst Aboriginal and Torres Strait Islander Australians: a scoping review

**DOI:** 10.1186/s40337-020-00346-9

**Published:** 2020-12-01

**Authors:** Adam Burt, Deborah Mitchison, Kerrie Doyle, Phillipa Hay

**Affiliations:** 1 School of Medicine, Western Sydney University, Campbelltown, Australia; 2 Translational Health Research Institute, School of Medicine, Western Sydney University, Campbelltown, Australia; 3 Centre for Emotional Health, Department of Psychology, Macquarie University, North Ryde, New South Wales Australia; 4 Campbelltown Hospital, SWSLHD, Campbelltown, Australia

**Keywords:** Feeding and eating disorders, Oceanic ancestry group, Aboriginal and Torres Strait islander, Diagnosis, Screening

## Abstract

**Background:**

Aboriginal and Torres Strait Islander Australians (Indigenous Australians) have poorer mental health compared to other Australians. Yet, there is a lack of research into mental disorders among this population, especially for eating disorders (ED), which are amongst the most lethal and debilitating mental disorders.

**Aim:**

We aimed to answer 2 questions: 1. What is the volume and content of literature on ED among Indigenous Australians? 2. Has a screening or diagnostic tool/instrument been developed for the assessment of ED amongst Indigenous Australians?

**Method:**

We conducted a scoping review of electronic databases (Pubmeb, Embase, PsychInfo, Proquest, Cochrane Library, Indigenous HealtInfoNet and Scopus), for studies addressing ED, body image, muscle dysmorphia, weight and shape concern among Indigenous Australians, as well as diagnostic and screening tools. All relevant studies were reviewed in full by 2 researchers. Narrative synthesis of the data was performed.

**Results:**

There is limited evidence for ED among Indigenous Australians, however, the evidence available strongly suggests that ED are more common among Indigenous Australians compared to other Australians. Eating disorders among Indigenous Australians are also associated with high levels of overvaluation of weight and shape. The increased risk of ED among Indigenous Australians was largely explained by factors such as poorer psychosocial wellbeing. No evidence was found for the existence of validated diagnostic or screening tools for ED in Indigenous Australians.

**Conclusion:**

The evidence suggests ED are common among Indigenous Australians, and there are no diagnostic or screening tools available to assist clinicians in assessing them. More research is required in this field, especially towards the development of a validated and culturally specific screening or diagnostic tool for ED among Indigenous Australians.

## Plain English summary

Indigenous-Australians are known to have poorer mental health compared to other Australians, yet there is a lack of research into mental disorders for Indigenous-Australians, especially for eating disorders. It is important to conduct research into eating disorders because they are among the most debilitating and lethal of all mental disorders.

We reviewed all the available research evidence addressing eating disorders amongst Indigenous-Australians, including for any tools used to assist in the screening and diagnosis of eating disorders among Indigenous-Australians. We included 7 research papers in the study. We found that there is limited evidence for eating disorders amongst Indigenous-Australians and the evidence available suggests that eating disorders are common amongst Indigenous-Australians, more common than amongst other-Australians. The most common eating disorders among Indigenous-Australians are those with overeating symptoms, including night eating syndrome amongst adolescents, and unspecified feeding and eating disorder with binge eating among Indigenous adults. Evidence from the studies we reviewed suggested that the increased risk of eating disorders among Indigenous-Australians was explained poorer social and psychological wellbeing. We found that there needs to be more research in this area as well as increased awareness of eating disorders amongst those caring for Indigenous-Australian patients.

## Introduction

### Mental health of Aboriginal and Torres Strait Islander Australians

Aboriginal and Torres Strait Islander Australians (Indigenous Australians) have poorer health, especially mental health, compared to other Australians. The Australian Burden of Disease Study is a large report produced by the Australian Government and provides data on the burden of fatal and non-fatal disease in the Australian population, including Indigenous Australians. According to the study, mental illness makes up the greatest burden of non-fatal disease amongst Indigenous Australians; the top 3 conditions being anxiety disorders, depressive disorders and alcohol use disorders [1]. Indigenous Australian adolescents also have a greater burden of conduct disorder, intentional self-harm and suicide, and 3-fold more hospital separations for psychosis and substance use disorders [1–3]. Indigenous Australians are also burdened with significant risk factors for physical and mental illness such as psychological distress. Numerous large studies have suggested up to a third of Indigenous Australians suffer high to very high levels of psychological distress, which is 3-fold higher than the rest of the Australian population [2, 4, 5]. The evidence suggests factors such as social and civic distrust, negative perception of neighborhoods, low socioeconomic status and an inability to access help from family have been found to explain the higher levels of psychological distress among Indigenous Australians [4]. Indigenous Australians are also a younger population than other Australians (21.8 years vs. 37.6 years) [1, 2, 6, 7]. Thus, many young Indigenous Australians are suffering debilitating mental illnesses in the prime of their lives and will likely live for many years with chronic mental illness and its associated medical and social complications. Aside from the significant psychosocial burden, these rates of psychological distress and mental disorders significantly increase the risk of medical conditions. Conditions such as eating disorders are also known to arise in young people [8] and are associated with physical comorbidities [9]. However, there is little consideration of these conditions in young Indigenous people.

### Eating disorders and rationale for a scoping review

Eating disorders are a group of mental disorders characterised by persistent disturbances in eating or eating related behaviour resulting in altered consumption or absorption of food, with accompanying psychopathology such as excessive body image concern and significant psychosocial impairment and distress [10]. Unique amongst mental health problems they are associated with significant physical morbidity involving many organ systems as a result of starvation, obesity or eating disorder related behaviours such as purging [9]. Eating disorders are also amongst the most life-threatening mental disorders, with a 6-fold increase in attempted suicide in those affected, and a persistently elevated risk of suicide after recovery [11–13]. Eating disorders are also very common, being prevalent in up to 22% of young Australians [14] and in 2011 accounting for 1.4% of the burden of non-fatal disease in Australia [15].

Despite the severity of eating disorders as mental illnesses and their prevalence in Australia, very little has been published on eating disorders in Indigenous Australians, and to date no review articles have been published specifically addressing eating disorders in this group. A systematic review aimed to identify the prevalence of psychiatric disorders among Indigenous Australians. The study included 17 articles, yet the authors found no prevalence data for several psychiatric disorders, including eating disorders [3]. It is unclear why there has been a lack of research into this area to date. A lack of identification of eating disorders and Indigenous Australians in epidemiological studies, and a lack of treatment seeking or identification of eating disorders, especially in primary care, may be reflected in the lack of data. There are similar issues in New Zealand where low rates of ethnicity reporting data and low inclusion rates of Māori in eating disorder research have left a gap in the evidence base, despite the equal prevalence of eating disorders between Māori and non- Māori New Zealanders [16, 17].

Identification of eating disorders relies on appropriate means of assessment, such as validated screening or diagnostic tools and interviews. This is especially important when they are used to screen or diagnose conditions in Indigenous Australians, as they differ from other Australians both in terms of culture, general health and wellbeing [18–20]. The adapted patient health questionnaire version 9 (aPHQ-9) is a recently developed, rigorously validated and culturally specific screening tool for depression in Indigenous Australians and is an exemplar in this regard. This tool was designed and validated with multiple Indigenous communities across Australia and is free to use [21]. However, a systematic review of diagnostic methods for psychiatric disorders in Indigenous Australians did not identify any culturally specific or validated diagnostic or screening tools for eating disorders among Indigenous Australians [22].

## Methods

We chose to conduct a scoping review of the literature in this emerging field to guide future research on this group of disorders for Indigenous Australians. Thus, our specific aim was to answer two questions: 1. What is the volume and content of literature on eating disorders among Indigenous Australians? 2. Has a screening or diagnostic tool/instrument been developed for the assessment of eating disorders amongst Indigenous Australians?

We developed a scoping review protocol (registered with The Open Science Framework (OSF), registration: osf.io/ptmdg. We utilised the framework for scoping reviews described by Arskey and O’Malley (2005): identify the research question, identify relevant studies, select studies, chart the data, collate and summarise the results, report, and consult with others in the field, in our case with Indigenous Australian academics and members of the Indigenous community [23]. We reported all items suggested by the Preferred Reporting Items for Systematic reviews and Meta-Analyses extension for Scoping Reviews (PRISMA-ScR) checklist [24]. The actual protocol used in the study was closely followed as registered on OSF.

### Search procedure

Given the aim, we opted to search widely, using multiple databases. We performed a systematic search of the electronic databases: Pubmeb, Embase, PsychInfo, Proquest, Cochrane Library, Indigenous HealtInfoNet and Scopus (for grey literature) using the search terms: [Indigenous Australian OR Aboriginal and Torres Strait Islander OR First Australians] AND [Eating Disorder OR Anorexia Nervosa OR Bulimia nervosa OR Binge eating OR body image OR body dysmorphia OR muscle dysmorphia OR weight/shape overvaluation]. Search terms such as body image, body dysmorphia and weight/shape overvaluation were included because they cover important areas of psychopathology in eating disorders and are areas of interest in research with Indigenous Australians. Manual searches of reference lists of articles used in this review were also conducted.

### Selection criteria

Studies included were: 1) prevalence surveys, review articles, clinical trials, case reports and observational studies published in English with no date limits, addressing Indigenous Australians with eating disorders or disordered eating, of all ages; 2) studies addressing eating disorder screening or diagnostic tools for Indigenous Australians; 3) inclusive of rural, remote, urban and metropolitan communities of Australia. The last search was conducted on 19th September 2020. Articles were screened by 1 reviewer, and if they met the selection criteria they were fully reviewed by 2 reviewers. Any disagreements were resolved by consultation with a 3rd reviewer.

### Data extraction and synthesis

**Fig. 1:**
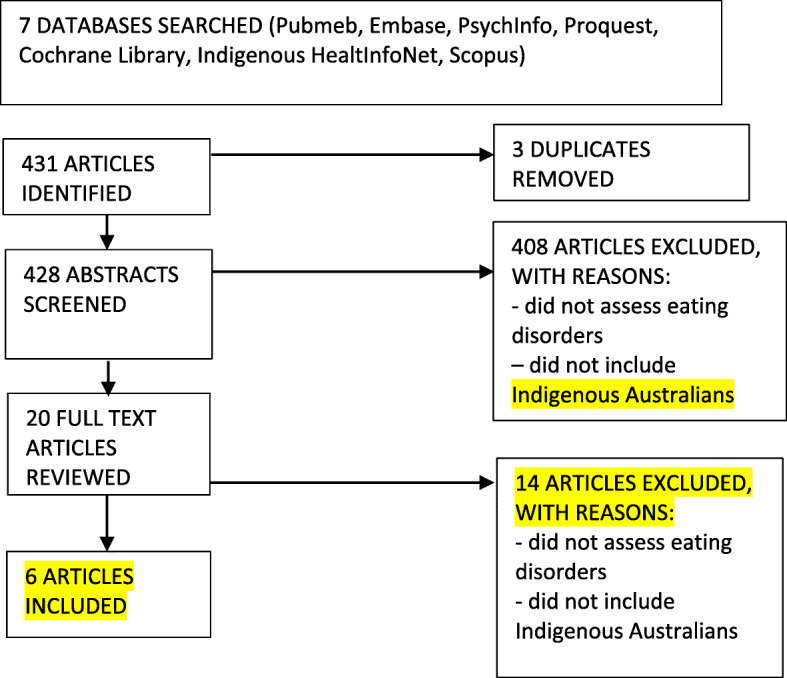
Flow diagram of study selection process

Figure 1 displays the article selection process. A table was developed for each research question extracting and summarising study characteristics:

1. *What is the volume and content of literature on eating disorders and disordered eating amongst indigenous Australians?*

Study details and characteristics: citation, country, context, participants, ethical considerations for Indigenous Australians. Results extracted from the study: type or quality of assessment of eating disorders/disordered eating (self-report, diagnostic or symptomatic, representative or convenience sample), what was assessed, what was reported in regard to epidemiology.

2. *Has a screening or diagnostic tool/instrument been developed for the assessment of eating disorders amongst indigenous Australians?*

Study details and characteristics: citation, country and context. Numbers, gender, response rate and ethnicity of participants. As well as ethical considerations for Indigenous Australians. Results extracted from the study (in relation to question 2: an assessment tool) such as quality of life domains assessed, number of items in the tool, details of psychometric validation of the tool.

A narrative synthesis of the data was performed, this type of synthesis is appropriate for studies scoping research such as this and is frequently used in scoping and systematic reviews [25, 26].

## Results

Of the 6 studies included, 5 were cross sectional, 1 was a systematic synthesis of population data. Two of the studies estimated eating disorders in Indigenous Australians to the diagnostic threshold level, and 2 estimated disordered eating features in Indigenous Australians. One study measured the impact of media messaging on body dissatisfaction in Indigenous compared other Australian adolescents. No studies were identified that directly assessed a screening or diagnostic tool for eating disorders in Indigenous Australians.

The 2 reviewers assessed all included articles for the reporting items in the protocol. The timeframe of the studies was from 2005 to 2020. All studies included both male and female participants. Details of the studies are found in Table 1 and attached online file.

**Table 1:** Articles included addressing eating disorders and eating disorder screening or diagnostic tools for Indigenous Australians

**Study Details and Characteristics**						
Study citation details	Mulders-Jones 2017 [27]	Hay 2012 [28]	Azzopardi 2018 [2]	Burt 2020 [29]	Burt 2020 [30]	McCabe 2005 [31]
Country	Australia	Australia	Australia	Australia	Australia	Australia
Context	Rural, urban and metropolitan	Rural, urban and metropolitan	Rural, urban and metropolitan	Rural, urban and metropolitan	Rural, urban and metropolitan	Urban (Melbourne)
1.Participants 2.Survey response rate	1.Male 2960 - Female 3081 - Total 6041, - Indigenous 118 2. Data from 2 surveys, 62.8% & 59.3%	1.Total 5926 -Female 3017 -Male 2909 Indigenous 155 -Female 91 -Male 64 2.Not given	360 papers sampling 10-24yo Indigenous adolescents.	1. Total 5068 -Male 3003 -Female 2065 Indigenous 402 -Female 143 -Male 259 -Did not disclose 80 2.Not given	1.Total 6041 Indigenous 92 -Female 53 -Male 42 2. Data from 2 surveys, 53.7% & 58.4%.	1.Total 100 Indigenous 50 -Female 25 -Male 25 12-16yo 2.Not given
Ethical considerations in regards to Indigenous Australian research.	Nil	Nil	Nil	Nil	Nil	Consultation from Aboriginal cooperatives and elders in the community where data gathered.
**Details**						
Quality or type of assessment: 1. self-report or interview 2. Diagnostic or symptoms. 3.Representative or convenience sample	1. Interview. 2. Symptoms. 3.Representative sample.	1.Interview. 2.Symptoms. 3.Representative sample.	N/A	1.Interview. 2.Diagnostic. 3.Representative sample.	1.Interview. 2.Diagnostic. 3.Representative sample.	1.Self report. 2. Symptomatic. 3. Convenience sample.
What was assessed?	Socioeconomic status and DE features.	Prevalence of DE features.	Population health data.	Prevalence of ED in Indigenous adolescents, and moderating effects of independent variables.	Prevalence of ED in Indigenous adults, and moderating effects of Independent variables.	BD using 5 subscales of body image and body image inventory.
What was reported in regards to epidemiology of DE/ED	There was an equal likelihood to report DE/ED features, regardless of identification as Indigenous. Those who did not report their Indigenous status reported higher levels of overvaluation of weight and shape.	Higher prevalence of DE/ED features amongst Indigenous.	Reported ED less common in Indigenous adolescents v. other Australians, more common in urban v. rural Indigenous adolescents (they did not identify a non-urban case). Population health data. Also reported more mood disorders, psychosis and psychological distress in Indigenous.	Indigenous Australians had higher prevalence of ED, mostly due to higher OSFED-NES in Indigenous Australians. OSFED-NES 7.14% (95%CI 4.81–10.49) of IA vs. 3.72% (95%CI 3.17–4.36) in other Australians. The greater NES was due to poorer Psychosocial QoL in Indigenous.	Indigenous Australians had a higher prevalence of ED, 27%. Most prevalent was UFED with binge eating. On logistic regression analysis independent variables: higher BMI, younger age and poorer MHRQoL were retained in model were the dependent variable was having an ED. ED were associated with high levels of overvaluation.	Indigenous Australians more BD than other Australians and undertook more strategies to decrease weight and increase weight and increase muscles. Indigenous girls had lower levels of BD than other Australian girls. Indigenous adolescents received less media messages although they had more impact.

*ED* Eating Disorder, *DE* Disordered Eating behaviour, *BD* Body Dissatisfaction

### Prevalence and correlates of eating disorders in the general population

A systematic synthesis of population data by Azzopardi et al., (2018) [2] aimed to report the health and wellbeing of Indigenous Australian adolescents based on 3 domains: health outcomes (mortality, avoidable mortality, disease and injuries), health risks (health behaviours and states) and sociocultural determinants (culture, family, physical wellbeing, education, employment etc). Data were included from 4 national surveys and 4 administrative data sets from national databases which were mapped to the 3 domains. No sample size estimate was provided. The authors suggested eating disorders were more common amongst other Australian adolescents compared to Indigenous Australian adolescents. The paper did not comment on community consultation, however, it did involve an Indigenous author.

The findings of the former study were contrasted by two more recent representative general population studies by Burt et al., (2020) [29, 30]. The authors estimated the diagnostic prevalence of eating disorders to be higher in adult (27%) and adolescent (29%) Indigenous Australians compared to non-Indigenous Australians. Indigenous Australian status did not explain the greater prevalence of eating disorders among the adult or adolescent groups. In the adult group the greater prevalence of eating disorders was associated with the higher body mass index (BMI), poorer mental health related quality of life (MHRQoL) and younger age of Indigenous Australians, Indigenous adults also experienced higher levels of overvaluation of weight and shape compared to other Australians. Similarly, in the adolescent group the higher eating disorder prevalence was largely explained by poorer psychosocial quality of life (Psychosocial QoL) experienced by the Indigenous Australian group, they did not however differ significantly in weight and shape concern compared to the other Australian adolescents. The most common eating disorder among Indigenous Australian adults was unspecified feeding or eating disorder with recurrent binge eating (UFED), among Indigenous adolescents the most common eating disorder was other specified feeding or eating disorder – night eating syndrome (OSFED-NES). Whilst neither study consulted the Indigenous community in the design of their research, both were written and informed by an Indigenous lead author. A limitation of the adult study by Burt et al. [30], was the small number of Indigenous participants (*n* = 92), and missing data due to a number of participants not disclosing their Indigenous status, albeit this was adjusted for in the analyses. Moreover, it is suggested the estimates may be conservative.

The contrasting findings between Burt et al. [29, 30], and Azzopardi et al. [2], may be because the latter was based on data from the national hospital morbidity and other databases for mental disorders. These data miss those who have not been admitted to hospital, which is almost certainly the majority of cases of eating disorders [32].

The former are further supported by two general population studies by Hay and Carriage (2012) and Mulders-Jones, Mitchison, Girosi and Hay (2017) who reported on prevalence of eating disorder behaviours. The authors found eating disorder behaviours to be of similar or increased frequency in Indigenous compared to non-Indigenous Australians. Neither reported on consultation with the Indigenous community, although the former included an Indigenous Australian author [27, 28].

When considered together, the findings from the adult and adolescent studies by Burt et al. [29, 30], and the former study by Hay and Carriage [28], suggest that eating disorders are more common among Indigenous Australians compared to other Australians, particularly eating disorders with overeating features such as UFED with recurrent binge eating and OSFED-NES.

### Body image concern

One study by McCabe et al., (2005) [31] measured the effect of media influences on the body dissatisfaction amongst 50 Indigenous Australian adolescents compared to a matched cohort of other Australian adolescents. The authors’ aims were to examine the extent of differences in body image and body change strategies and awareness of media messages to change weight and shape for Indigenous and non-Indigenous adolescents from urban areas, and to examine the extent to which these media messages predicted body image and body change strategies in these groups. Overall, Indigenous adolescents engaged in more activities to lose weight, increase weight and increase muscles than non-Indigenous adolescents; however in sex-stratified analyses, Indigenous girls reported less body image dissatisfaction compared to non-Indigenous girls. The authors suggest the Indigenous adolescents may have been less aware of the media messages’ impact on their behaviour or were more reluctant to report that media messaging impacted their behaviour. The high levels of weight and shape concern among Indigenous adolescents found in this study accords with the findings of research by Burt et al. [29, 30].

The authors of this study consulted local Indigenous elders in preparation for the research.

### Screening or diagnostic tools

No studies were identified that directly assessed a diagnostic or screening tool for eating disorders.

## Discussion

There is limited evidence addressing mental disorders amongst among Indigenous Australians, especially for eating disorders, which is both a major finding and a limitation of the research literature. The majority of evidence available on eating disorders amongst Indigenous Australians comes from 3 cross sectional papers by members of this research team: the adult and adolescent studies by Burt et al., (2020) [29, 30], the study by Hay and Carriage, (2012) [28], supported by the body image study by McCabe et al., (2005) [31]. When these studies are considered together with well demonstrated significant burden of mental illness [1] and mental illness risk factors [4] in the Indigenous population, they strongly suggest that eating disorders are more common among Indigenous Australians compared to other Australians. This suggestion is supported by the consistency in the types of disorders (those with overeating features) prevalent in the Indigenous community, the explanatory variables (poorer psychosocial wellbeing) for those disorders and the prominent psychopathology (overvaluation of weight and shape), supported across multiple studies and in different age groups. We suggest that within the Indigenous population there exists a relatively larger pool of younger people with poorer mental health, high levels body image concern that ostensibly does not diminish with adulthood, and severe socioeconomic disadvantage, leaving that population more vulnerable to developing an eating disorder. These findings of elevated general psychosocial impairment also accord with other studies that have found an increased risk for a range of mental disorders among Indigenous Australians, that is, this risk is not specific to eating disorders [33, 34].

No studies were identified in this review that directly assessed a diagnostic or screening tool for eating disorders among Indigenous Australians. One systematic review aimed to examine the cultural validity of the methods used to diagnose psychiatric disorders among Indigenous Australians, however, it not include any articles directly assessing eating disorders, thus it was not included in this review [22].

The implications of these findings are the need for greater awareness of eating disorders among those who work in Indigenous health, among parents of Indigenous children, as well as schoolteachers who may be among the first to notice eating disorder behaviours in young people. Further research is needed to assess the uptake of treatment for eating disorders among Indigenous Australians to guide future research into effective treatments as well as identification of eating disorders in this population. Indigenous Australians are culturally and linguistically different to other Australians, even in areas where the Indigenous Australian population speak English almost exclusively. There are very few Indigenous Australian healthcare workers, especially those likely to come into contact with undifferentiated patients with eating disorder symptoms such as Nurses, Psychologists, General Practitioners and Psychiatrists. The assessment and treatment of eating disorders is a specialised field, and not always easily accessible for patients in Australia. Moreover, the majority of clinicians who treat Indigenous Australian patients are not eating disorder experts, therefore, the absence of a culturally specific, validated screening tool is of directly clinical relevance and importance and should be a priority for researchers in order to assist those who see Indigenous Australian patients. This field of research, including qualitative research conducted within an Indigenous paradigm and within the Indigenous community should aim to understand the nature of eating disorders so that effective treatments may also be developed, such as psychotherapies.

## Conclusions

The evidence available strongly suggests that eating disorders are more common amongst Indigenous Australians compared to other Australians and are associated with high levels of overvaluation of weight and shape. To the best of our knowledge no research has been published addressing diagnostic or screening instruments to help detect eating disorders amongst Indigenous Australians. More research is required in this field, especially towards the development of a validated and culturally specific screening or diagnostic tool/instrument for eating disorders amongst Indigenous Australians, ideally conducted in collaboration with Indigenous communities and Indigenous researchers.

## Data Availability

All data reported is published and in the public domain.

## Abbreviations

ABS: Australian Bureau of Statistics
AMS: Aboriginal Medical Service
aPHQ-9: Adapted Patient Health Questionnaire
BED: Binge Eating Disorder
BMI: Body Mass Index
BN: Bulimia Nervosa
CI: Confidence Interval
df: Degrees of freedom
DSM-5: Diagnostic and Statistical Manual of Mental Disorders 5th edition
ED: Eating Disorder
IA: Indigenous Australian
HOS: Health Omnibus Survey
n: Number of participants
NES: Night Eating Syndrome
OR: Odds Ratio
OSFED: Other Specified Feeding or Eating Disorder
UFED: Unspecified Feeding or Eating Disorder
YLD: Years Lived with Disability.
